# Detection of Arthropod-Borne Bacteria and Assessment of MALDI-TOF MS for the Identification of Field-Collected Immature Bed Bugs from Mauritania

**DOI:** 10.3390/insects14010069

**Published:** 2023-01-11

**Authors:** Jacques Sevestre, Mohamed Aly Ould Lemrabott, Jean-Michel Bérenger, Adama Zan Diarra, Ali Ould Mohamed Salem Boukhary, Philippe Parola

**Affiliations:** 1 Institut Hospitalo-Universitaire Méditerranée Infection, 19-21 Boulevard Jean Moulin, CEDEX 05, 13385 Marseille, France; 2 IRD, AP-HM, SSA, VITROME, Aix Marseille University, 13005 Marseille, France; 3 Unité de Recherche Génomes et Milieux, Faculté des Sciences et Techniques, Université de Nouakchott, Nouakchott BP 880, Mauritania

**Keywords:** *Cimex hemipterus*, *Cimex lectularius*, bed bugs, MALDI-TOF MS, Mauritania

## Abstract

**Simple Summary:**

Bed bugs are hematophagous insects that have been described as a cause of nuisance since ancient times. Two species are known to parasitize humans: *Cimex lectularius*, prevalent in temperate areas, and *C. hemipterus*, also known as the tropical bed bug. A global increase in infestation has been observed in recent decades. However, data is lacking concerning species distribution, which may be affected by population movements (e.g., travelers, migrants). Moreover, although these insects are not known to harbor and transmit infectious agents to humans, continuous monitoring of their infection status is part of active surveillance. We analyzed bed bug specimens collected from beddings in Mauritania, and assessed MALDI-TOF MS as an identification tool for immature specimens. Moreover, we also conducted bacterial detection in each specimen. Overall, our findings supported MALDI-TOF MS as being a robust tool allowing species level identification of immature *Cimex* specimens.

**Abstract:**

Human infestations by bed bugs have upsurged globally in recent decades, including in African countries, where recent reports pointed out an increase in infestation. Sympatric dwelling has been described for two species of bed bug parasitizing humans: *Cimex hemipterus* (the tropical bed bug) and *C. lectularius*. Identification of these two species is based on morphological characteristics, and gene sequencing, and may also rely on Matrix-Assisted Laser Desorption Ionization Time-Of-Flight Mass Spectrometry (MALDI-TOF MS). The present work aimed to assess whether MALDI-TOF MS was applicable for species level identification of immature stages of *Cimex*. Arthropods were collected in domestic settings in Nouakchott, Mauritania. Identification used morphological keys and MALDI-TOF MS identification was assessed for immature stages. Quantitative PCR and sequencing assays were used to detect arthropod-associated bacteria in each specimen. A total of 92 arthropods were collected, all morphologically identified as *C. hemipterus* (32 males, 14 females and 45 immature stages). A total of 35/45 specimens produced good quality MALDI-TOF MS spectra. Analysis allowed species level identification of all immature *C. hemipterus* after their spectra were entered into our in-house MALDI-TOF MS arthropod spectra database. Molecular screening allowed detection of *Wolbachia* DNA in each specimen. These results suggested that MALDI-TOF MS is a reliable tool for species level identification of *Cimex* specimens, including immature specimens. Future studies should assess this approach on larger panels of immature specimens for different *Cimex* species and focus on the precise staging of their different immature developmental stages.

## 1. Introduction

True bugs of the *Cimex* genus are globally distributed hematophagous arthropods, some of which are known to have parasitized humans since ancient times [1]. Two species preferentially feed on humans: *Cimex lectularius*, the common bed bug, and *Cimex hemipterus*, the tropical bed bug [2]. Although the role of bed bugs as vectors of infectious diseases to humans has been discussed but not yet proven, infestation may result in serious psychological issues in infested individuals [2]. Moreover, the prevalence of bed bug infestation has dramatically increased worldwide in recent decades [2]. Thus, increasing knowledge concerning these arthropods, their potential role as vectors, and the proposing of adequate control measures are issues more than ever on the agenda.

The simultaneous presence of *C. lectularius* and *C. hemipterus* has already been described in several tropical and subtropical areas worldwide, and notably in Eastern and Western Africa, in which sympatric dwelling has previously been described [3,4,5,6,7]. However, to date, no data is available concerning the *Cimex* sp. fauna in Mauritania, West Africa.

Population movements have an impact on the health and management of health systems, notably concerning vector-borne diseases in the countries of transit and destination [8]. Mauritania’s particular geographical location, between North and sub-Saharan Africa, makes this country a temporary destination for migrants seeking to reach Europe, and, thus, represents an interesting interplay in migrating diseases. Its potential role in the dynamics of parasitizing arthropods and vector-borne diseases deserves to be studied. Indeed, in recent decades, several vector-borne diseases with potentially devastating effects on humans and animals have been detected in Mauritania [9].

Prior to any control measures, identification of medically important arthropods at the species level is mandatory. This is generally done by morphological identification, which is helped by the use of magnifying tools, along with dichotomic identification keys [10]. However, this method requires entomological expertise and available documentation, and may be impeded by the specimen’s condition (for example, if body parts are missing) [11]. Moreover, even when performed by trained experts, misidentification may occur [12]. Species level identification may also accurately rely on molecular biology methods, which are, however, time consuming, and may engage high running costs [12].

In recent decades, Matrix Assisted Laser Desorption Ionization Time-Of-Flight Mass Spectrometry (MALDI-TOF MS) has been successfully proposed as an identification method, notably concerning medically important arthropods [11]. In particular, this approach has been recently applied for species level identification of adult stages of various *Cimex* species [13,14]. However, to our knowledge, this identification technique has never been assessed on immature stages within the *Cimex* genus.

The objective of the present work was to assess MALDI-TOF MS as an identification tool over field-collected immature stages of *Cimex* sp. specimens, collected in domestic settings in Mauritania. Secondarily, all specimens (adult, as well as immature, stages) were screened for potential arthropod-borne bacteria using molecular methods.

## 2. Material and Methods

### 2.1. Sampling Sites

**Figure 1:**
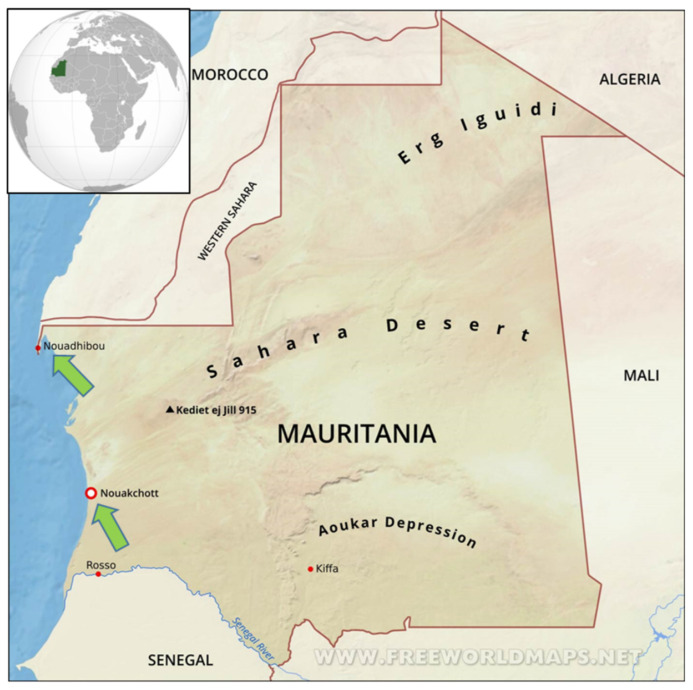
Representation of Mauritania, West Africa. The two localities in which the bed bugs were collected in domestic settings, between September 2021 and October 2021, are indicated with green arrows (from North to South: Nouadhibou and Nouakchott).

Bed bug sampling was conducted simultaneously in October 2021 in two locations in Western Mauritania, Nouadhibou and Noaukchott (Figure 1). Nouadhibou is the economic capital city, located between 20°56′5.843″ North latitude and 17°2′20.317″ West longitude. It has a desertic climate, with an average annual temperature of 21.2 °C and a rainfall record below 50 mm per year. Nouakchott is the administrative capital of the country, located between 18°8′50.725″ North latitude and 15°56′35.733″ West longitude, on the edge of the Atlantic Ocean. The climate of Nouakchott is tropical arid, with an average rainfall comprised between 30 to 50 mm per year.

### 2.2. Bed Bugs: Collection, Morphological Identification and Dissection

Bed bug specimens were collected in beddings in domestic settings in Nouakchott and Nouadhibou, Mauritania (Figure 1). Adult and immature stages were collected and preserved in 70% ethanol, before being shipped to the Institut Hospitalo-Universitaire Méditerranée Infection in Marseille, France, for further analyses (shipment authorized by the French Ministry for Agriculture under the reference ER18-2021). All specimens were observed using an AxioZoom V16 stereomicroscope (Zeiss, Oberkochen, Germany) at a magnification of ×56. Identification at the species level used an identification key applied to *Cimex* adult species [10]. In the absence of an identification key for immature *Cimex* specimens when the present work was initiated, species level identification of immature stages relied on notable morphological features applicable for adult specimens, including pronotum width, bristle length and density as well as the specimen’s general coloration [10]. Some specimens were also selected for electron microscopy, using a TM4000 Plus scanning electron microscope (Hitachi, Tokyo, Japan), as previously described [14]. Following morphological identification, each specimen was dissected using a sterile surgical blade. The immature specimens’ heads were separately kept for MALDI-TOF MS analysis, as previously described [13]. Secondarily, the most distal part of the abdomen of each specimen was dissected and kept as material for DNA extraction. The rest of each specimen’s body was kept as a backup sample at −80 °C.

### 2.3. MALDI-TOF MS Identification

Immature specimens’ heads were incubated at 37 °C overnight in order to facilitate ethanol evaporation [15]. Following incubation, each head was crushed for two minutes in an extraction mix solution containing 20 µL of 70% formic acid and 20 µL of 50% acetonitrile, as previously described [7]. After a brief spin, protein extracts were spotted on the MALDI-TOF target plate, left to dry and covered with 1 µL of a CHCA matrix suspension, as described [13]. Protein mass spectra were obtained using a Microflex LT MALDI-TOF Mass Spectrometer (Bruker Daltonics, Bremen, Germany), with parameters set as described [13]. Protein mass profiles were visualized using the Flex Analysis v.3.3 and ClinProTools 2.2 software (Bruker Daltonics). Only good quality spectra were retained for MALDI-TOF MS identification. Good quality spectra were defined as reproducible, without baseline noise and with peak intensity >3000 arbitrary units [7]. These spectra were secondarily queried against our in-house arthropod database (DB) using the MALDI Biotyper v.3.0 software (Bruker Daltonics) [11,16]. This database notably contains reference spectra for many different arthropods accumulated over the years, including 45 mosquito species and 59 tick species, as well as reference spectra for adult and immature stages of *C. hemipterus* and *C. lectularius*, from various geographical origins (*C. hemipterus* from Kenya and Senegal; *C. lectularius* from France, Germany, United Kingdom, Sweden) [13]. The reliability of identification was estimated through Log Score Values (LSVs) ranging from 0 to 3. When MALDI-TOF MS analysis produced low-quality spectra, or when LSV values were below 1.8 arbitrary units, specimens were retained for sequencing in order to confirm species level identification. Following molecular identification, high-quality MS spectra from two immature specimens selected among our study sample were added to the pre-existing database, before our samples were queried against the upgraded database.

### 2.4. DNA Extraction and Molecular Identification of Cimex sp.

The distal part of each bed bug specimen’s abdomen was transferred in a 1.5 mL tube with 180 µL of G2 lysis buffer and 20 µL of proteinase K (Qiagen, Hilden, Germany) and incubated overnight at 56 °C. DNA extraction was performed using an EZ1 DNA Tissue Kit (Qiagen), according to the manufacturer’s protocol, as previously described [7]. The DNA from 13 *Cimex* specimens was subjected to standard PCR and sequencing using a thermal cycler automaton (Applied Biosystems, Foster City, CA, USA) with primers targeting the 18S rDNA gene [17]. The DNA from laboratory-bred *C. lectularius* was used as a positive control. The sequences obtained were assembled and identified, as previously described [7].

### 2.5. Detection and Identification of Bacteria in Cimex sp.

The DNA extract from each specimen was subjected to five different quantitative PCR (qPCR) assays for the detection of *Borrelia*, *Rickettsia*, *Bartonella*, *Coxiella burnetii* and *Wolbachia*, as previously described [18]. The reaction mixture was composed of 5 µL of the template’s DNA and 15 µL of the qPCR mix previously described [19]. Amplification was performed using a LightCycler 480 thermal cycler automaton (Roche Diagnostics, Basel, Switzerland). Specimens were considered positive if target sequence DNA was detected with a Cycle Threshold (Ct) value under 36. The DNA from our laboratory-cultured strains of *Rickettsia montanensis*, *Bartonella elizabethae*, *Coxiella burnetii*, and *Borrelia crocidurae* were used as positive controls, as previously described [7]. For *Wolbachia* detection, a positive DNA extract previously sequenced as *Wolbachia pipientis* was used as positive control. DNA extracted from a laboratory-bred specimen of *Rhipicephalus sanguineus* s.l. tick was used as a negative control. When DNA from *Wolbachia* was detected from a *Cimex* specimen, bacterial species level identification was performed by gene sequencing, following standard PCR amplification of a fragment of *16SrDNA*, as previously described [7].

## 3. Results

### 3.1. Specimen Collection and Morphological Identification

**Figure 2:**
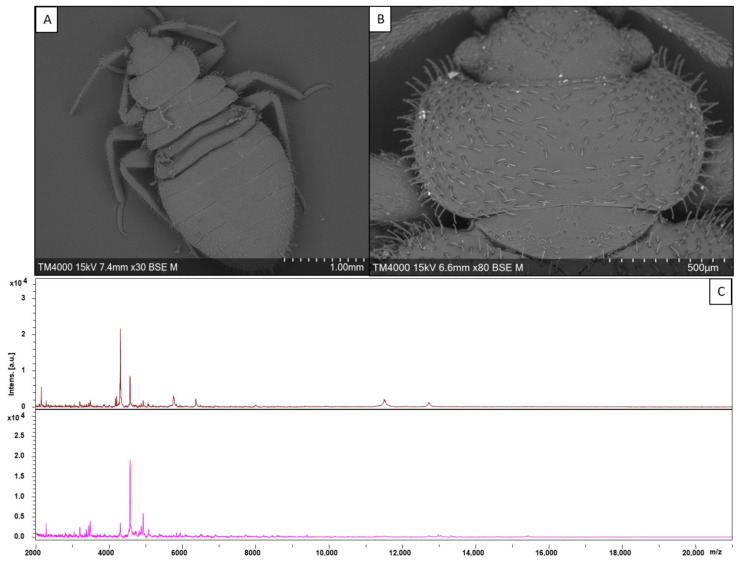
(**A**) Electron microscopy photograph of an immature specimen of *Cimex hemipterus.* (**B**) Electron microscopy photograph of the pronotum of an immature specimen of *C. hemipterus*. (**C**) Representation of species-specific Matrix-assisted Laser Desorption-Ionization Time-Of-Flight Mass Spectrometry Mass Spectra obtained through analysis of two immature specimens of *C. hemipterus*, collected in Mauritania between September 2021 and October 2021. The *y* axis refers to intensity and is expressed in arbitrary units ([a.u.]). The *x* axis refers to the mass to charge ratio (*m*/*z*).

A total of 92 specimens were collected within beddings in two domestic apartments in Nouakchott and Nouadhibou, Mauritania, between October and November 2021 (Figure 1). Following reception in Marseille, 91 specimens were identified as *Cimex hemipterus*, including 45 immature stages (49.4%), 32 males (35.1%) and 14 females (15.3%). Approximately half of the specimens were engorged (49/92; 53.2%). One specimen could not be morphologically identified due to poor conservation state. Among our study sample, two immature specimens were selected for scanning electron microscopy imaging (Figure 2).

### 3.2. MALDI-TOF MS Identification

Among the 45 immature specimens, a total of 43 were submitted to MALDI-TOF MS analysis. Two specimens were selected for electron microscopic study. Among the 43 specimens tested subjected to MALDI-TOF MS analysis, a total of 35 specimens produced good quality spectra (Figure 2). When entered into our in-house arthropod MALDI-TOF MS database, a total of 35/35 specimens were identified as *C. hemipterus*, congruently with morphological identification. Precisely, 29 specimens were identified as *C. hemipterus* with LSVs over 1.8 (1.809 to 2.238), and 6 specimens were identified as *C. hemipterus* with LSVs ranging from 1.58 to 1.786. All these MALDI-TOF MS spectra matched with higher LSV with reference spectra obtained from heads of field-collected adult *C. hemipterus* originating from Senegal, which were preserved dried (following a quick immersion in 90% EtOH at the moment of collection) [7]. Lower LSV were obtained with reference spectra obtained from cephalothorax of fresh immature specimens of *C. hemipterus* originating from Kenya and reared in our laboratory. Secondarily, our pre-existing database was upgraded with spectra originating from two of our samples. Querying of our spectra against the upgraded database resulted in more reliable identification scores, with 35/35 (100%) of the specimens identified as *C. hemipterus*. Precisely, 3/35 specimens retrieved LSVs between 1.967 and 1.990, and the remaining (32/35) specimens were identified with LSVs >2.

### 3.3. Molecular Identification of Cimex Specimens

A total of 14 immature specimens morphologically identified as *C. hemipterus* were subjected to molecular identification through sequencing of the 18S rDNA gene: 8/14 because of obtention of low-quality spectra through MALDI-TOF MS analysis, and 6/14 because of obtention of Log Score Values <1.8 (e.g., ranging from 1.58 to 1.786) after spectra were entered into our in-house arthropod database. BLAST analysis allowed identification of *C. hemipterus* for all the 14 specimens subjected to molecular identification, with identification percentages ranging from 99.2% to 100% (GenBank: MN056507), confirming the identification.

### 3.4. Detection and Identification of Bacteria in Cimex Specimens

All the 92 specimens’ DNA extracts were submitted to specific qPCR assays for detection of *Borrelia*, *Rickettsia*, *Bartonella*, *Coxiella burnetii* and *Wolbachia*. All tested specimens were positive for the *Wolbachia* qPCR, with Ct values ranging from 29.93 to 37.82 (average: 31.04, median: 30.91). All DNA samples tested negative for the other bacterial detection assays. Four DNA samples (obtained from two adults and two immature stages) were selected for *Wolbachia* 16S rDNA sequencing. Following amplification and sequencing, good quality sequences were obtained for the four specimens. BLAST analysis of the four sequences showed that they were 99.2% and 99.7% similar to the corresponding sequences of *Wolbachia pipientis* (GenBank: MN123078), and other *Wolbachia* sp., including a sequence obtained from *C. lectularius* bed bugs originating from Serbia (MH618380.1), and from *C. hemipterus* from Senegal (Personal Data).

## 4. Discussion

The present work focused on MALDI-TOF MS identification of field-collected immature *Cimex* specimens originating from Nouakchott and Nouadhibou, Mauritania. To the best of our knowledge, the present work represents the first proteomic and molecular investigation concerning bed bugs in this West-African country.

Bed bugs nowadays represent a global nuisance issue, as corroborated by recent scientific literature and rather important media coverage [2,20,21]. Indeed, this global problem has not spared the African continent, which recently experienced a tangible reemergence of bed bug infestations, notably among refugee camps and hosting facilities, but also in households [7,21,22]. It is assumed that insecticide resistance in *Cimex* species could act as a key driver for its expansion in this continent [7,22,23]. Thus, increasing knowledge concerning the biology, lifestyle, possible vector competence and capacity of these arthropods, and the implementation of reliable identification methods, appears crucial nowadays [7].

In our study sample, all bed bugs specimens were identified as *C. hemipterus*. This species is generally associated with tropical settings, although it has already been reported in sub-tropical and temperate areas [2,7]. Taking into account the recent global spread of *Cimex* species parasitizing humans, the geographical distribution of both species could shift in the following years, highlighting the need for deployment of robust species level identification techniques in order to effectively monitor their distribution [2,7].

MALDI-TOF MS has proved to be a robust tool for species level identification of a wide range of arthropods impacting human and animal health [11,12]. Indeed, this identification tool may help circumvent some of the difficulties specific to morphological identification (e.g., the need for entomological expertise, available documentation, well-preserved specimens, etc.) and molecular identification (e.g., specific automatons and reagents, as well as availability and reliability of sequences deposited on GenBank) [11,12]. The reliability of the MALDI-TOF MS approach resides in creation of a specific arthropod spectra database, originating from specimens unambiguously identified at the species level [11]. Reference spectra originate from a specific body compartment, from which the choice depends on the compartment’s susceptibility to allow the obtaining of reproducible, species-specific spectra [11]. In past years, the use of this technique for *Cimex* species level identification has been assessed in regard to, and successfully applied to, various species of medical and veterinary interest [7,13,14]. In the present work, we demonstrated, for the first time, that MALDI-TOF MS was a reliable tool for the identification of ethanol-preserved *Cimex* specimens, and also that it was applicable for the identification of immature stages of this genus.

Given the availability of recognized morphological identification criteria between adult stages of *C. hemipterus* and *C. lectularius*, and the previously published work assessing MALDI-TOF MS identification for adult stages of the *Cimex* genus, only immature stages were retained for MALDI-TOF MS identification in the present work [7,13,14]. Indeed, at the time these methods were performed, no morphological identification key was available for staging the different immature developmental cycles of *Cimex hemipterus* species [10]. However, a morphological identification key for staging of the immature stages of *C. hemipterus* collected from Ghana has been recently proposed [24]. This staging key proposition relies on morphometric analysis of the antennal segments in immature specimens. As noted by the authors, antennal length appears to be distinctive in each *C. hemipterus* stage, whereas other morphological characters seem to overlap among developmental stages, as assessed by means of Principal Component Analysis (PCA) by the authors [24]. This staging key could be further assessed among *C. hemipterus* specimens originating from other geographical areas. Furthermore, the applicability of MALDI-TOF MS to effectively stage immatures should also be assessed, with the help of laboratory-bred colonies, for instance. Ongoing works in our institute are currently investigating MALDI-TOF MS applicability to discriminate the different immature developmental stages of *Cimex* species.

Another interesting part of our results resides in the fact that the spectra obtained from our sample (e.g., heads of immatures) matched more closely with spectra obtained from field-collected adult specimens of *C. hemipterus* originating from Senegal, even though our arthropod spectra database also contained reference spectra from immature stages of *C. hemipterus* and *C. lectularius*. However, previous studies focusing on MALDI-TOF MS identification of adult *C. hemipterus* specimens used heads as a study compartment, whereas reference spectra for immature stages in our in-house database were obtained from cephalothoraxes [7,13]. In the present work, our proteomic identification results could be explained by subtle differences in protein profiles obtained from each compartment, possibly due to the cephalothorax’s protein content. Indeed, choice of a single compartment is necessary to obtain reproducible spectra [11]. The following factors are known to impact MS spectra, as previously hypothesized in earlier studies: the specimen’s preservation method, its geographic origin, the microbiota impact, or the presence of blood meal remnants [11,25,26]. In this context, we believe that the choice of using the heads as the study compartment could better explain matching with reference spectra obtained from heads of adult field-collected specimens, regardless of the developmental stage. Moreover, as Mauritania borders Senegal, we hypothesize that a geographic factor might also participate in the better identification of our specimens, as previously discussed (9).

In the present work, bacterial screening and identification only allowed detection of *Wolbachia* DNA in our study specimens. These bacteria act as endosymbionts for numerous arthropods and have been detected in specimens originating from multiple geographical locations [7,27,28].These microorganisms have been reported to be involved in the insect’s development, reproductive potential and blood meal digestion. [27,28]. Recently, Ndiaye et al. reported detection of different *Wolbachia* species in field-collected *Cimex* specimens originating from Senegal [7]. The author’s approach used a panel of three different genes to discriminate different *Wolbachia* species in their study sample, which allowed detection of *Wolbachia massiliensis* and *W. pipientis*, as well as *Wolbachia* sp, along with phylogenetic analysis of their likelihood [7]. In our study sample, *Wolbachia* DNA was detected from all specimens, and sequencing from four of our specimens showed that these sequences were close to the corresponding sequences of *W. pipientis*, and *Wolbachia* sp., with similar identification percentages. Even though detection of *Wolbachia* is common among cimicids, more data is necessary concerning the different species that could be detected in bed bugs to better understand their respective impacts on their arthropod hosts [7,28].

No other potentially vector-borne bacterial DNA was detected from our *Cimex* study sample. This data was in accordance with previously published studies assessing infection status with vector-borne bacteria for field-collected *Cimex* specimens [2,7,13]. Indeed, even though some experimental infection studies have shown a potential vector competence of *Cimex* specimens for vector-borne infectious agents, such as *Borrelia recurrentis*, *Bartonella quintana* and *Trypanosoma cruzi*, their role as infectious disease vectors in “real life” has never yet been demonstrated [2,29,30]. Continuous studying of infection status of *Cimex* sp., as well as future studies evaluating their vector competence for other vector-borne microorganisms should enable better knowledge of their potential as infectious disease vectors.

In the end, even taking into account the relative low number of specimens assessed, these results suggest that MALDI-TOF MS could be a robust tool for identification of immature stages of *Cimex hemipterus*. These results shed a promising light on effective identification and monitoring of *Cimex* populations. Indeed, MALDI-TOF MS has established itself as a current reference method for identification of medically important microbes in clinical microbiology laboratories [31]. Besides its robustness, rapidity and cost-effectiveness as a part of the workflow in clinical microbiology, this technique may also be used for research purposes, including arthropod identification, without additional costs [11,12]. The possibility of including both routine identification of medically important microorganisms and arthropod identification is particularly interesting in developing countries, notably on the African continent, in which availability of this tool continues to expand [32,33].

## Data Availability

The data presented in this study are openly available in IHU at https://doi.org/10.35081/4PVF-XB11 (accessed on 10 January 2022). Moreover, sequences have been deposited in GenBank under accession numbers OP898543-OP898556.
